# Mixed etiology COVID-19 associated acute rhinosinusitis caused by two *Aspergillus* species

**DOI:** 10.1016/j.amsu.2022.103365

**Published:** 2022-02-11

**Authors:** Payam Tabarsi, Somayeh Sharifynia, Mihan Pourabdollah Toutkaboni, Zahra Abtahian, Mohammad Rahdar, Arefeh Sadat Mirahmadian, Atousa Hakamifard

**Affiliations:** a Clinical Tuberculosis and Epidemiology Research Center, National Research Institute of Tuberculosis and Lung Diseases (NRITLD), Shahid Beheshti University of Medical Sciences, Tehran, Iran; b Chronic Respiratory Diseases Research Center, National Research Institute of Tuberculosis and Lung Diseases (NRITLD), Shahid Beheshti University of Medical Sciences, Tehran, Iran

**Keywords:** Aspergillosis, Fungal infections, *Aspergillus*, rhino‐sinusitis, *SARS-CoV-2*, *Aspergillus flavus*, *Aspergillus niger*

## Abstract

**Introduction:**

Acute invasive fungal rhino‐sinusitis (AIFR) is a life-threatening infection that is mostly found in immunocompromised patients with serious morbidity and mortality. Recently, reports of AIFR have also emerged among SARS-CoV-2 infected patients.

**Case presentation:**

A 50-year-old diabetic woman, previously diagnosed with COVID-19 pneumonia, was presented to the hospital with left facial pain on day 12 after discharge. Paranasal sinuses computed tomography was performed and according to the mucosal thickening in both maxillary sinuses and ethmoidal air cells, the patient underwent functional endoscopic sinus surgery (FESS) and necrosis were observed. The histopathologic examination revealed mycelium with septation suspected to *Aspergillus* and the culture was consistent with *Aspergillus flavus* and also *Aspergillus niger***.** We reported a case of COVID-19 associated AIFR with two combined *Aspergillus* species from Iran. The patient received liposomal amphotericin B, which then switched to voriconazole combined with aggressive surgical debridement of necrotic tissues with a clinically favorable outcome.

**Conclusion:**

Mixed etiology AIFR can influence the outcome. However, further investigation is required upon this new threat.

## Highlights

• Acute invasive fungal rhino‐sinusitis (AIFR) is a life threatening infection mostly found in immunocompromised patients.
• Reports of AIFR have emerged among *SARS-CoV-2* infected patients.
• Mixed etiology AIFR can also influence the outcome and further studies are required upon this new threat.

## 1 Introduction

The number of reported cases of invasive fungal infections with *Aspergillus* species in COVID-19 patients are on an increasing trend. *Aspergillus* species is a distributed filamentous fungus worldwide. They are believed to be the leading cause of life-threatening invasive fungal infections, such as pneumonia and sinusitis in COVID-19 patients, especially in those with risk factors, including prolonged neutropenia [1]. There are numerous reports on co-infections with fungal pathogens, but two fungal co-infections are rarely reported, especially in acute rhinosinusitis as a complication of COVID-19. Herein; we present a case of acute invasive fungal rhinosinusitis (AIFR) with mixed proven fungal etiology (*Aspergillus niger* and *Aspergillus flavus*). Our case report was written according to SCARE guidelines [2].

## 2 Case report

**Fig. 1:**
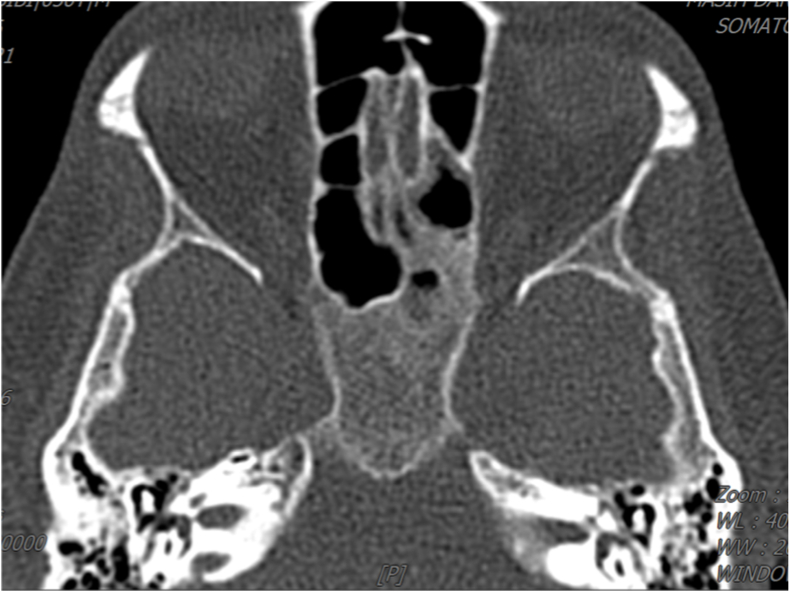
Mucosal thickening is seen in both maxillary sinuses and ethmoidal air cells.

**Fig. 2:**
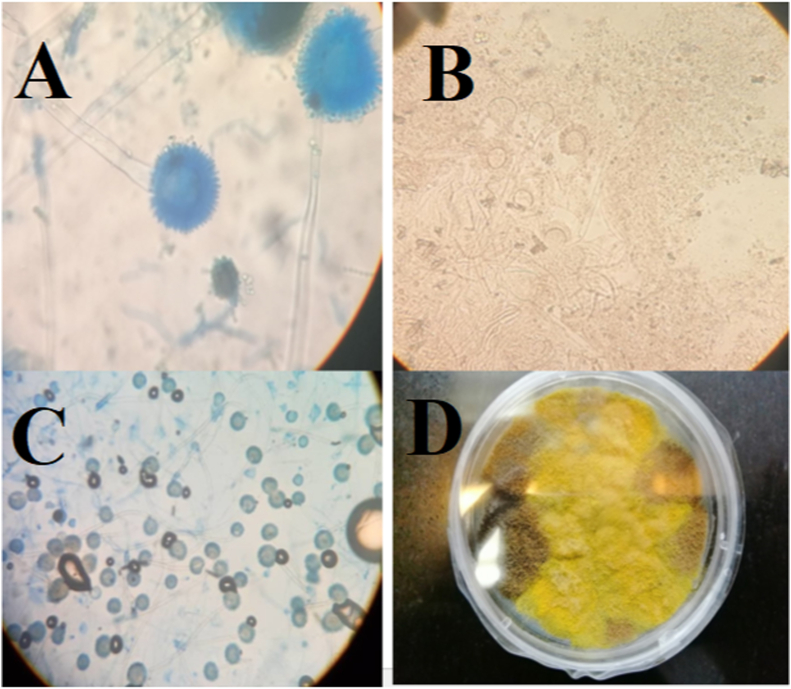
Culture phenotypically identified as *Aspergillus flavus* and *Aspergillus niger*.

**Fig. 3:**
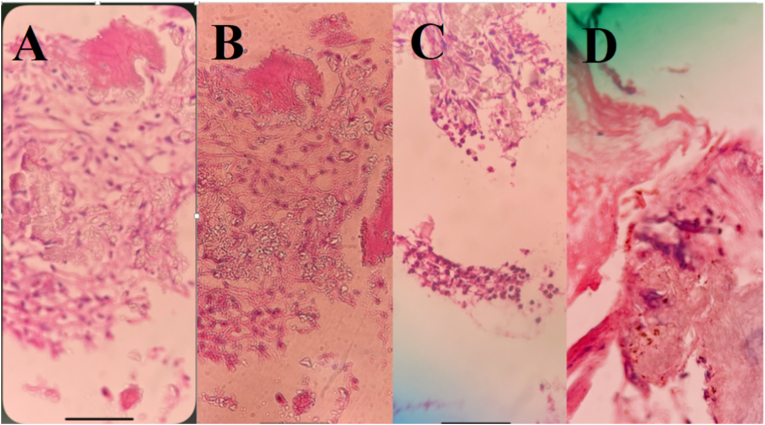
Several calcium oxalate crystals, indicative of aspergillosis (A), calcium oxalate crystals are highlighted with low light (B), aggregation of polymorphonuclear leukocytes in favor of acute inflammation (C), one degenerated hyphae that seems to invade bone accompanied by calcium oxalate crystals (D).

A 50-year-old diabetic woman with a six-day history of fever (38.3 °C), myalgia, dry cough and severe dyspnea (SpO2 was 86% - room air) was presented to the emergency department. She had a medical history of type 2 diabetes mellitus treated with an oral hypoglycemic agent and history of hypertension. RT-PCR confirmed SARS-Cov-2 diagnosis. High-resolution chest CTs obtained at admission revealed areas of ground-glass opacification. The patient received supportive oxygen therapy with nasal cannula in addition to remdesivir at a dose of 200 mg on day 1 and 100 mg/d for 5 days and methylprednisolone 60 mg every 12 hours for three days. After three days, the methylprednisolone was switched to dexamethasone 4 mg/d continued for 7 days. The patient's condition improved over 15 days, and she was discharged with SpO2 = 93%, afebrile, and improved cough. Following 12 days, the patient presented to our hospital with a history of left facial pain. At admission, physical examination showed a hemodynamically and respiratory stable condition. The initial laboratory data were as follows: white blood cells (WBC) 12,500/μl, platelet (PLT) 300,000/μl, hemoglobin (Hb) 11 gr/dl, creatinine (Cr) 0.8, and C reactive protein (CRP) 19 mg/dl. In the physical examination, extra oral swelling over the left side of her face and numbness of the left side of her upper lip was observed. Subsequently, computed tomography of sinuses and lungs were performed (Fig. 1). According to the mucosal thickening in both maxillary sinuses and ethmoidal air cells, the patient underwent functional endoscopic sinus surgery (FESS) and severe involvement and necrosis were observed. Microscopic examination revealed mycelium with septation suspected to *Aspergillus* species. The culture was consistent with *Aspergillus* spp phenotypically resemble *Aspergillus flavus* and *Aspergillus niger* (Fig. 2). In pathology specimen of the left maxillary sinus acute on chronic osteomyelitis associated with many calcium oxalate crystals, as byproducts of *Aspergillus* spp, and occasional degenerated fungal hyphae were also observed (Fig. 3). Empirical treatment with liposomal amphotericin B (5 mg kg−1 d−1) was initiated. According to the results of pathology and the diagnosis of proven aspergillosis with two species, intravenous voriconazole 400 mg was administered every 12 hours on day 1 and 300 mg was prescribed every 12 hours for the following days. The patient was discharged with no signs of necrosis and therapy switched to oral voriconazole. After one month of follow-up, she is still healthy.

## 3 Discussion

Herein, we reported the case of COVID-19 associated AIFR with two mixed *Aspergillus* species; *Aspergillus flavus* and *Aspergillus niger* in Iran, and one of the few cases reported worldwide. Invasive fungal infections were observed in COVID-19 patients and were considered as the leading cause of death in 25%–73.7% of patients; hence, it is necessary to take into account the probability of fungal co-infections in patients with COVID-19 [3]. AIFR is a life-threatening and time-sensitive condition, which is most often found in immunocompromised patients with subsequent high morbidity and mortality (18%–80%), despite improvements in medical and surgical management protocols [4,5]. In the study by El‐Kholy et al., the most common symptoms of patients with AIFR were headache and facial pain (75%), facial numbness (66.7%), ophthalmoplegia and visual loss (63.9%). Additionally, in the mentioned study, mycological analysis revealed mucormycosis and aspergillosis was diagnosed in 77.8% and 30.6% of patients, respectively [6].

There are several reports on fungal co-infections with COVID-19; meanwhile, mixed fungal co-infections, with more than one aspergillus species, have been rarely reported. In the report by Costache et al., the authors reported a case of COVID associated pulmonary aspergillosis (CAPA) with *Aspergillus* section *Fumigati* and *Aspergillus* section *Flavi* from Romania with favorable clinical outcomes [7]. Nasir N et al. described a fatal case of CAPA with mixed aspergillus etiology; *Aspergillus flavus* and *aspergillus fumigatus* [8].

There are some reports on fungal co-infections in AIFR. Zayet et al. reported a case of cerebro-rhino-orbital mucormycosis and aspergillosis coinfection in a patient with diabetes mellitus with complete recovery after amphotericin B deoxycholate then voriconazole in combination with surgical debridement [9]. It was assumed that a combination of risk factors, including COVID-19 per se, diabetes mellitus, and corticosteroid treatment, had led to mixed infections in the presented case.

Several mechanisms in COVID-19 patients have been proposed, which predispose the patient to invasive fungal infections, with the endothelial damage and immune dysfunction being the most important causes. COVID-19 infection can disrupt the immune system by affecting T lymphocytes, especially cytotoxic T cells (CD8^+^) and helper T cells (CD4^+^). The latter consists of helper T cells that have a specific cytokine profile, such as IFN-α and TNF-α, which result in the activation of macrophages and neutrophils that modulate antifungal immunity [[10], [11], [12]].

Tissue invasion, with a filamentous fungus through histopathological examination of biopsy, provides a diagnosis of confirmed invasive fungal infections and positive culture of *Aspergillus* from the specimen, requires for proven diagnosis of invasive aspergillosis [13,14]. In our case, calcium oxalate crystals, accompanied by tissue invasion of fungal hyphae in addition to positive culture, confirmed the diagnosis of invasive aspergillosis caused by *Aspergillus flavus* and *Aspergillus niger*.

Co-infections with fungal pathogens are identified in *SARS-CoV-2*. Mixed fungal pathogens have become another serious threat in the treatment of patients with COVID-19, which should not be neglected. Mixed etiology of AIFR can also influence the outcome. Nevertheless, further research is required in this regard.

## Ethical approval

This research was approved by the ethics committee of Shahid Beheshti University of Medical Sciences and written informed consent was obtained from the patient.

## Sources of funding

No funding.

## Author contributions

Payam Tabarsi, Somayeh Sharifynia, Mihan Pourabdollah Toutkaboni, Zahra Abtahian, Mohammad Rahdar, Arefeh Sadat Mirahmadian and Atousa Hakamifard acquired data, analyzed and interpreted the data.

Somayeh Sharifynia and Mihan Pourabdollah Toutkaboni; identified *Aspergillus* species.

Atousa Hakamifard wrote the first draft of the manuscript.

All authors have read and approved the final manuscript.

## Registration of research studies

**1.** Name of the registry: **Not applicable (case report)**

2. Unique Identifying number or registration ID:

3. Hyperlink to your specific registration (must be publicly accessible and will be checked).

## Guarantor

Atousa Hakamifard: atousahakamifard@sbmu.ac.ir.

## Consent

Written informed consent was obtained from the patient.

## Provenance and peer review

Not commissioned, externally peer-reviewed.

## Disclosure of interest

The authors declare that they have no competing interest.

## Declaration of competing interest

No conflict of interest.
